# Effects of Cyclic Mechanical Stretch on the Proliferation of L6 Myoblasts and Its Mechanisms: PI3K/Akt and MAPK Signal Pathways Regulated by IGF-1 Receptor

**DOI:** 10.3390/ijms19061649

**Published:** 2018-06-02

**Authors:** Shaoting Fu, Lijun Yin, Xiaojing Lin, Jianqiang Lu, Xiaohui Wang

**Affiliations:** School of Kinesiology, Shanghai University of Sport, Shanghai 200438, China; 1610302017@student.sus.edu.cn (S.F); 1620302032@student.sus.edu.cn (L.Y.); 1711516005@student.sus.edu.cn (X.L.)

**Keywords:** cyclic mechanic stretch, L6 myoblasts, proliferation, IGF-1, PI3K/Akt, MAPKs

## Abstract

Myoblast proliferation is crucial to skeletal muscle hypertrophy and regeneration. Our previous study indicated that mechanical stretch altered the proliferation of C2C12 myoblasts, associated with insulin growth factor 1 (IGF-1)-mediated phosphoinositide 3-kinase (PI3K)/Akt (also known as protein kinase B) and mitogen-activated protein kinase (MAPK) pathways through IGF-1 receptor (IGF-1R). The purpose of this study was to explore the same stretches on the proliferation of L6 myoblasts and its association with IGF-1-regulated PI3K/Akt and MAPK activations. L6 myoblasts were divided into three groups: control, 15% stretch, and 20% stretch. Stretches were achieved using FlexCell Strain Unit. Cell proliferation and IGF-1 concentration were detected by CCK8 and ELISA, respectively. IGF-1R expression, and expressions and activities of PI3K, Akt, and MAPKs (including extracellular signal-regulated kinases 1 and 2 (ERK1/2) and p38) were determined by Western blot. We found that 15% stretch promoted, while 20% stretch inhibited L6 myoblast proliferation. A 15% stretch increased IGF-1R level, although had no effect on IGF-1 secretion of L6 myoblasts, and PI3K/Akt and ERK1/2 (not p38) inhibitors attenuated 15% stretch-induced pro-proliferation. Exogenous IGF-1 reversed 20% stretch-induced anti-proliferation, accompanied with increases in IGF-1R level as well as PI3K/Akt and MAPK (ERK1/2 and p38) activations. In conclusion, stretch regulated L6 myoblasts proliferation, which may be mediated by the changes in PI3K/Akt and MAPK activations regulated by IGF-1R, despite no detectable IGF-1 from stretched L6 myoblasts.

## 1. Introduction

Appropriate exercise increases [1,2,3], while excessive exercise or overtraining decreases the skeletal muscle mass and strength in animals and human [4,5]. Appropriate exercise leads to satellite cell activation and transformation to myoblasts, and promotes proliferation and differentiation of myoblasts, as well as fusion into multinucleated myotubes in vivo, thus inducing skeletal muscle hypertrophy [6,7]. However, the underlying mechanisms of appropriate exercise-induced muscle hypertrophy have not been fully elucidated, let alone the mechanisms of excessive exercise on the decrease of muscle mass.

Mechanical stretches on skeletal muscles play an important role in exercise-regulated skeletal muscle hypertrophy and injury/remodeling, so exogenous mechanical stretch was used to mimic exercise stimulation in vitro. For the crucial effects of skeletal muscle satellite cells and myoblasts on muscle hypertrophy, repair and regeneration [8], these cells, such as primary satellite cells and mouse C2C12 myoblasts receiving different mechanical stretches are good models to clarify the molecular mechanisms of myogenesis responses to exercise in vitro.

Mounting evidence, including our previous work, demonstrated that appropriate cyclic mechanical stretches with multiple deformations (10%, 15%, and 17%), frequency (0.25, 0.5, and 1 Hz), and durations (1 h, 2 h) promoted the proliferation of primary satellite cells and C2C12 myoblasts [9,10], and inhibited their differentiation into myotubes. However, few studies reported the influence of overstretch on cell proliferation and differentiation, and despite its important significance in regulating excessive exercise-induced muscular adaptation, the limited literature has reported that when the magnitude of cyclic mechanical stretch increased up to 20%, the proliferation of C2C12 myoblasts [9] and rat aortic smooth muscle cells [11] were inhibited, and even induced apoptosis in myoblasts [12]. However, studies about stretching on another myoblast cell line- rat L6 myoblasts are rare, and the effects of mechanical stretch on the proliferation and differentiation of L6 myoblasts remain unknown.

Phosphoinositide 3-kinase (PI3K)/Akt activation fulfils crucial roles in regulating cell proliferation and protein synthesis. Mechanical stretch-induced vascular remodeling process was through PI3K/Akt activation [13]. In C2C12 myoblasts, the pro-proliferation of 15% mechanical stretch was also through PI3K/Akt activation [9], furthermore, PI3K inhibitor LY294002 inhibited the proliferation of C2C12 myoblasts [9] and adult bovine myoblasts [14]. 

Three subsets of mitogen-activated protein kinases (MAPKs), extracellular signal-regulated kinases 1 and 2 (ERK1/2), p38 MAPK (p38), and c-Jun N-terminal kinase (JNK), are vital pathways that transfer extracellular signals into cells, and are closely associated with the proliferation and differentiation of cells [15,16]. Growing evidence points to the important role of MAPKs in mechanical stretch regulated proliferation, differentiation, and protein synthesis of skeletal muscle cells. For example, 10% of cyclic mechanical stretch stimulated the proliferation and inhibited the differentiation of bovine satellite cells via activation of ERK1/2 and of C2C12 myoblasts via activations of p38 and ERK1/2 [9]; cyclic stretch promoted the protein synthesis of C2C12 by increasing the activities of p38 and ERK1/2, and JNK responsive to 30 min of 5% or 15% stretch [17], and by activating ERK1/2 and p38 responsive to 24 h of 5% stretch [18]. 

It is well established that the most common upstream signal molecule of PI3K/Akt is insulin-like growth factor (IGF-1), which plays an important role in both the proliferation and differentiation of myoblasts. Exogenous IGF-1 not only induced myoblast proliferation in vitro in a dose-dependent manner, but also increased satellite cell number in the skeletal muscle of embryonic chickens [19]. The primary effects of IGF-1, including activating PI3K/Akt pathway, are mediated by binding to the IGF-1 receptor (IGF-1R), a widely expressed cell surface heterotetramer. Mechanical stretch enhanced the proliferation of venous smooth muscle cells [20], and primary cardiac fibroblasts via activation of IGF-1R/PI3K/Akt pathway. 

As for the association of MAPK with IGF-1 and IGF-1R, it has been reported that IGF-1 and IGF-1R affected cell proliferation and differentiation via ERK1/2 and p38 in human dental pulp stem cells [21] and via ERK1/2 in perivascular adipocyte [22]. Our previous work indicated that the pro-proliferation of 15% stretch and anti-proliferation of 20% stretch on C2C12 myoblasts were mediated by upregulating and downregulating IGF-1-induced activations of PI3K/Akt and MAPKs (p38 and ERK1/2), respectively. 

Therefore, the purpose of the present study is to clarify whether 15% and 20% cyclic mechanical stretches modulate the proliferation of rat L6 myoblasts, and whether the effects of stretches are related to the expressions and activities of PI3K/Akt and MAPKs (p38 and ERK1/2) regulated by IGF-1/IGF-1R.

## 2. Results

### 2.1. Effects of Cyclic Mechanical Stretch on the Proliferation of L6 Myoblasts

**Figure 1:**
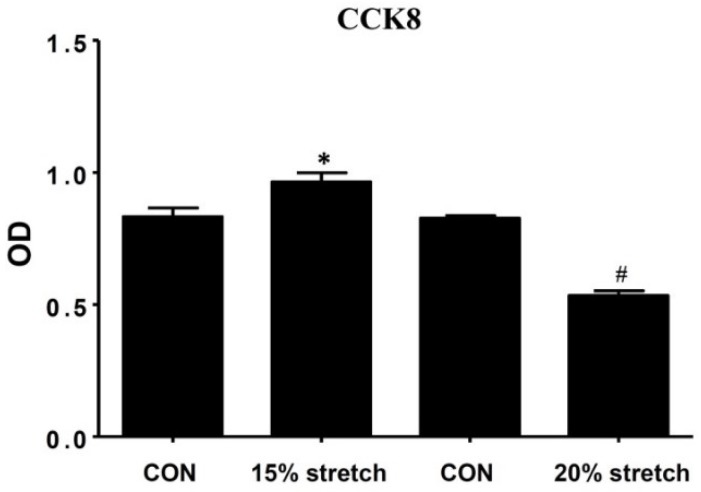
Effects of cyclic mechanical stretch on the proliferation of L6 myoblasts. L6 myoblasts were seeded onto flexible-bottomed 6-well plates coated with type I collagen at 1 × 10^5^/mL and incubated for 24 h, then the cells were subjected to 15% or 20% cyclic mechanical stretch at 0.5 Hz for 6 h. At 24 h after stretch finished, the proliferation of L6 myoblasts was detected by CCK8. The optical density (OD) results from three independent experiments were compared (mean ± SD, *n* = 3) (* and # indicated *p* < 0.05 vs. relative unstretched control (CON)).

Effects of 15% and 20% cyclic mechanical stretches on the proliferation of rat L6 myoblasts were detected, and we found that the proliferation of L6 myoblasts was significantly increased by 15% stretch for 6 h, while decreased by 20% stretch for 6 h compared with relative control (CON), respectively (Figure 1).

### 2.2. Effects of Cyclic Mechanical Stretch on the Expressions and Activities of PI3K/Akt and MAPKs (p38 and ERK1/2) in L6 Myoblasts

**Figure 2:**
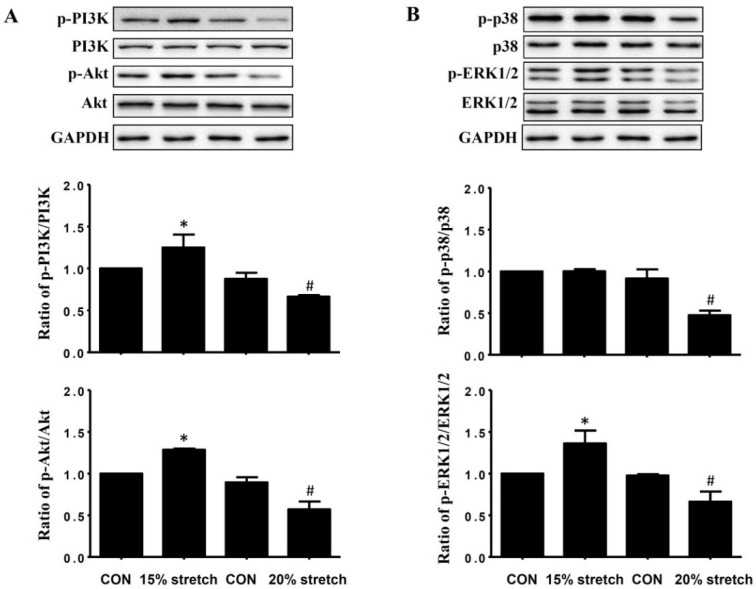
Effects of cyclic mechanical stretch on the expressions and activities of PI3K, Akt, and MAPKs (p38 and ERK1/2) in L6 myoblasts. The protein levels of PI3K, Akt, p38, and ERK1/2, as well as the activities of PI3K, Akt, p38, and ERK1/2 (reflected by the ratios of p-PI3K/PI3K, p-Akt/Akt, p-p38/p38, and p-ERK1/2/ERK1/2, respectively) were detected by Western blot at 24 h after stretch finished, and glyceraldehyde-3-phosphate dehydrogenase (GAPDH) was used as an internal control. The experiments were repeated three times, and one representative result of Western blot was shown in the upper of (**A**,**B**), and the normalized numbers (expressed as fold of control) from three independent experiments were compared, as shown in the bottom of (**A**,**B**) (mean ± SD, *n* = 3) (* and # indicated *p* < 0.05 vs relative CON). The quantitative results of the protein levels of PI3K, Akt, p38, and ERK were not shown due to no difference between stretched and unstretched cells.

Both 15% and 20% stretch did not change the protein levels of PI3K (PI3K/ glyceraldehyde-3-phosphate dehydrogenase (GAPDH)) and Akt (Akt/GAPDH) in L6 myoblasts compared with unstretched control (CON). However, the activities of PI3K (ratio of p-PI3K/PI3K) and Akt (ratio of p-Akt/Akt) were significantly increased in 15% stretched L6 myoblasts, while significantly decreased by 20% stretch (Figure 2A).

As shown in Figure 2B, neither 15% nor 20% stretch had an effect on the protein levels of p38 and ERK1/2 compared with CON, but ERK1/2 activity (ratio of p-ERK1/2/ERK1/2) was significantly increased in 15% stretched L6 myoblasts, while it was decreased in 20% stretched cells. For p38 activity (ratio of p-p38/p38), it was unaltered by 15% stretch, but significantly declined by 20% stretch.

### 2.3. Pro-Proliferative Effect of 15% Mechanical Stretch on L6 Myoblasts Was Reversed by PI3K/Akt and ERK1/2 Inhibitors Rather Than p38 Inhibitor

**Figure 3:**
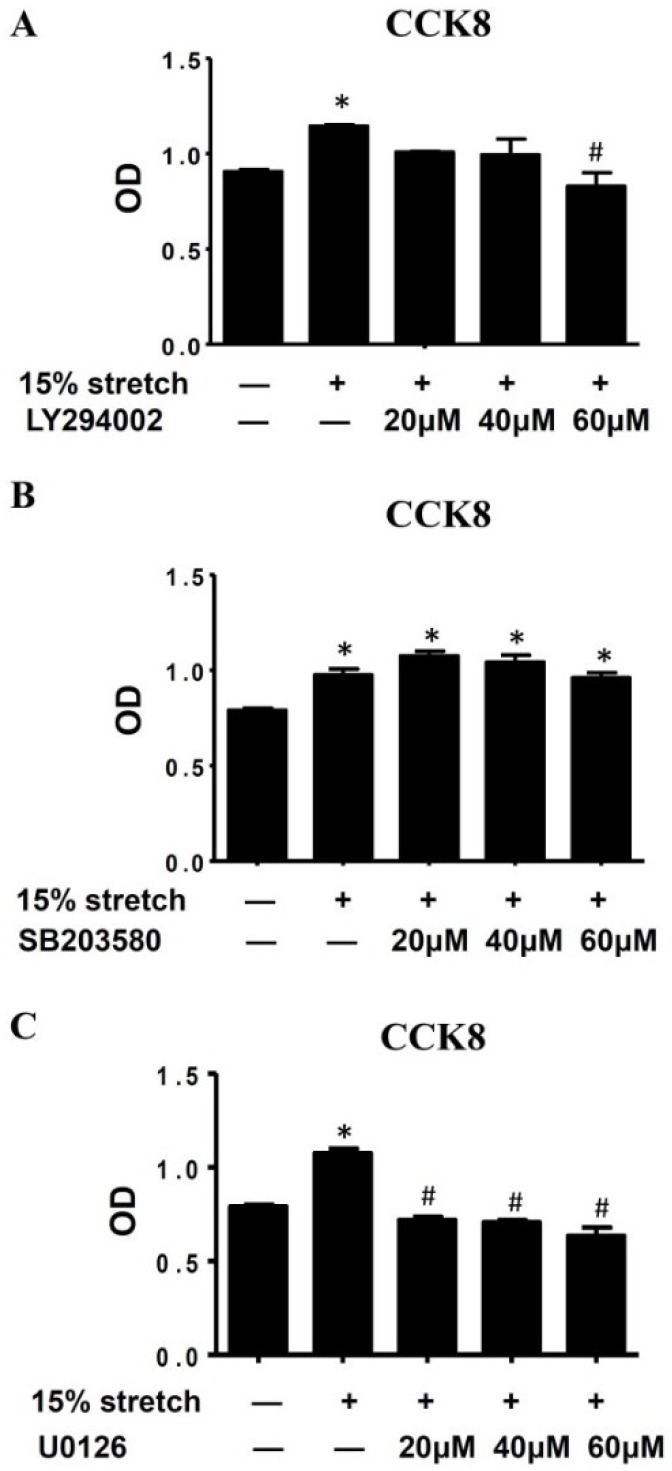
Pro-proliferative effect of 15% mechanical stretch on L6 myoblasts was reversed by the inhibitors of PI3K/Akt (**A**) and ERK1/2 (**C**) rather than p38 (**B**) inhibitor. L6 myoblasts were seeded at 1 × 10^5^/mL density, and cultured for 24 h, then treated with (**A**) PI3K specific inhibitor LY294002 (20 µM, 40 µM, and 60 μM), or (**B**) p38 specific inhibitor SB203580 (20 µM, 40 µM, and 60 μM), or (**C**) ERK1/2 specific inhibitor U0126 (20 µM, 40 µM, and 60 µM) for 2 h prior to 15% cyclic mechanical stretch. At 24 h after stretch finished, the proliferation of L6 myoblasts was determined by CCK8. The OD results from three independent experiments were compared (mean ± SD, *n* = 3) (* *p* < 0.05, vs. CON; # *p* < 0.05 vs. 15% stretch).

To verify the roles of PI3K/Akt and MAPKs (p38 and ERK1/2) in 15% cyclic mechanical stretch-induced proliferation of L6 myoblasts, specific inhibitors of PI3K (LY294002), p38 (SB203580) and ERK1/2 (U0126) were used before 15% stretch, to inhibit the activities of PI3K, p38, and ERK1/2 of L6 myoblasts, respectively. As shown in Figure 3, PI3K inhibitor (60 μM) and ERK1/2 inhibitor (20 μM), rather than p38 inhibitor (20 μM, 40 μM and 60 μM), blockaded the pro-proliferative effect of 15% stretch on L6 myoblasts.

### 2.4. Effects of Cyclic Mechanical Stretch on IGF-1 Secretion and IGF-1R Protein Level of L6 Myoblasts

**Figure 4:**
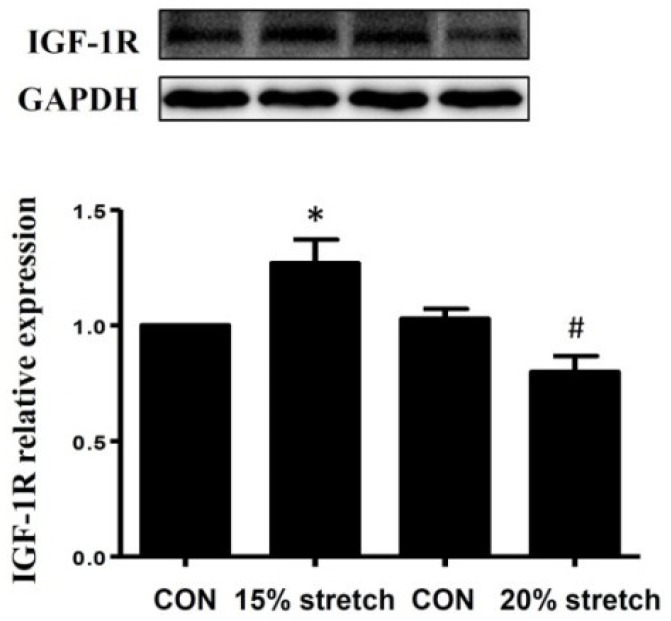
Effects of cyclic mechanical stretch on IGF-1R protein level of L6 myoblasts. The protein level of IGF-1R was detected by Western blot at 24 h after stretch finished, and GAPDH was used as an internal control. The results were representative of at least three independent experiments. The values were expressed as fold of CON and represent means ± SD (* and # indicated *p* < 0.05 vs. relative CON).

To verify whether the role of PI3K/Akt and MAPKs (p38 and ERK1/2) in L6 myoblasts proliferation was associated with IGF-1/IGF-1R, IGF-1 secretion and IGF-1R protein level were detected by ELISA and Western blot, respectively. Despite no detectable IGF-1 was secreted from stretched L6 myoblasts, IGF-1R protein level was increased by 15% stretch while decreased by 20% stretch, as shown in Figure 4.

### 2.5. IGF-1 Recombinant Peptide Alleviated 20% Stretch-Induced Proliferation Inhibition of L6 Myoblasts, Accompanied with the Increase of IGF-1R Protein Level as Well as Enhancements of PI3K/Akt and MAPKs (p38 and ERK1/2) Activities

**Figure 5:**
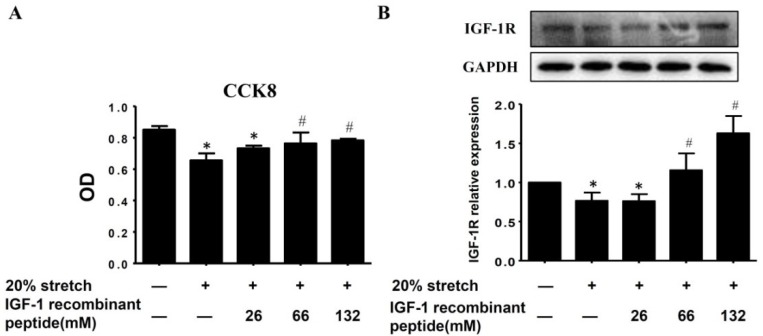

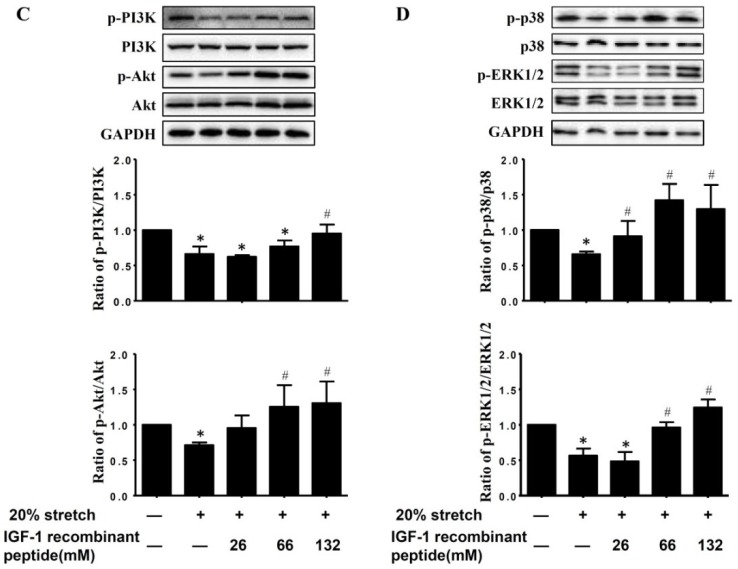
IGF-1 recombinant peptide alleviated 20% stretch-induced proliferation inhibition of L6 myoblasts (**A**), accompanied with the increase of IGF-1R protein level (**B**) as well as enhancements of PI3K/Akt (**C**) and MAPKs (p38 and ERK1/2) (**D**) activities. L6 myoblasts were treated with different concentrations (26, 66, or 132 mM) of IGF-1 recombinant peptide 1 h prior to 20% cyclic mechanical stretch. Culture medium was replaced with fresh medium immediately after stretch finished and cultured for a further 24 h, then the proliferation of L6 myoblasts was determined by CCK8 (Figure 5A), and the protein level of IGF-1R (Figure 5B), as well as the expressions and activities of PI3K/Akt ((Figure 5C) and MAPKs (p38, ERK1/2) (Figure 5D) were detected by Western blot. All experiments were repeated three times (mean ± SD, *n* = 3). The OD results of three independent experiments were compared, and are shown in Figure 5A. The results of Western blot were representative of at least three independent experiments, as shown in Figure 5B–D. The values are expressed as fold of CON and represent means ± SD. * *p* < 0.05 vs. CON; # *p* < 0.05 vs. 20% stretch. The quantitative results of the protein levels of PI3K, Akt, p38, and ERK were not shown due to no difference between stretched and unstretched cells.

To confirm the role of decreased IGF-1 in the proliferation inhibition of L6 myoblast and its association with the expressions and activities of PI3K/Akt, p38, and ERK1/2, IGF-1 recombinant peptide was added into culture medium at different concentrations (26, 66, or 132 mM) before 20% stretch. As shown in Figure 5A, IGF-1 recombinant peptide (66 mM, 132 mM) reversed the proliferation inhibition of L6 myoblasts induced by 20% stretch, which was accompanied with the increase of IGF-1R protein level (Figure 5B), as well as the enhancements of PI3K/Akt, p38, and ERK1/2 activities (not the protein levels of PI3K/Akt, p38, and ERK1/2), which indicated that 20% stretch-induced proliferation inhibition of L6 myoblasts was likely to be associated with the inhibitions of PI3K/Akt, p38, and ERK1/2 activities resulting from declined IGF-1R.

## 3. Discussion

Cellular responses to mechanical stimulus vary not only with parameters of applied mechanical stretch, such as magnitude, frequency, and duration, but also depending on the type of cells being stretched, so on the basis of pro-proliferation of 15% mechanical stretch (6 h at 0.5 Hz) and anti-proliferation of 20% stretches (6 h at 0.5 Hz) on mouse C2C12 myoblasts from our previous study [9], one purpose of the present study was to detect the influence of similar stretches on rat L6 myoblasts. We found that 15% and 20% mechanical stretches lasting for 6 h at 0.5 Hz promoted and inhibited the proliferation of rat L6 myoblasts respectively, similar to C2C12 myoblasts, which suggested the universality of stretch-induced proliferation changes in myoblasts. To our knowledge, this is the first report about the effects of mechanical stretch on the proliferation of L6 myoblasts. 

PI3K/Akt and MAPKs (p38 and ERK1/2) exert considerable roles on exercise-regulated skeletal muscle mass by regulating cell cycle progression. It has been reported that PI3K/Akt, a master cascade signal in satellite cells or myoblasts, regulated G1/S cell cycle progression by altering the levels and activities of cell cycle regulators [14,19]. Similarly, ERK1/2 activation by low frequency pulsed electromagnetic field was also shown to be crucial for the transition from G1 to S phase in C2C12 myoblasts [23], and the inhibition of p38 activation hindered satellite cells entering the cell cycle. By regulating cell cycle progression, the activated PI3K/Akt, p38, and ERK1/2 fulfilled the pro-proliferation of appropriate stretch on stretched cells. For example, 5% stretch at 0.1 Hz induced a remarkable increase of proliferative activity in smooth muscle cells via PI3K [24]; 18% mechanical stretch for 6 h also activates PI3K/Akt in aortic smooth muscle cells [13]; and 10% stretch cyclic mechanical stretch stimulated C2C12 myoblasts proliferation via prolonged activation of p38 MAPK while stimulating bovine satellite cell proliferation through activation of ERK1/2. Our previous study demonstrated the pro-proliferative effect of 15% stretch at 0.5 Hz was mediated by the activations of PI3K/Akt, p38, and ERK1/2 in C2C12 myoblasts [9]. In the present study, we found that 15% cyclic mechanical stretch promoted the proliferation of L6 myoblasts, accompanied with PI3K/Akt and ERK1/2 activations, but not p38 activation, furthermore, PI3K and ERK1/2 inhibitors, instead of p38 inhibitor, attenuated 15% stretch-induced pro-proliferation of L6 myoblasts, which indicated the pro-proliferation of 15% stretch on L6 myoblasts was mediated by PI3K/Akt and ERK1/2 activations, rather than p38 activation. These results were not totally the same as those of C2C12 in p38 activation, which might be associated with the different cell type. Similarly, the difference between C2C12 and L6 myoblasts was also found in response to extracellular stimuli, such as small molecule pharmacology and hormone [25]. 

As mentioned in the introduction, IGF-1/IGF-1R signaling pathway plays a key role in regulating skeletal muscle mass. Resistance training, the most useful treatment for the loss of muscle mass and strength in aging people, was determined to upregulate IGF-1 and IGF-1R expression levels [26], and skeletal muscle-specific IGF-1 overexpression promoted myofiber hyperplasia [27]. It is worth mention that IGF-1 is upstream of PI3K/Akt and MAPKs (p38 and ERK1/2) in regulating cell proliferation, for example, ERK1/2 and PI3K/AKT pathways both mediate the stimulatory effect of IGF-1 on myoblast proliferation [14], and IGF-1 prevents simvastatin-induced myotoxicity in C2C12 myotubes through IGF-1R-Akt [28], and IGF-I induced proliferation of L6 myoblasts via the mediation of IGF-1R regulated activations of PI3K/Akt and ERK1/2 pathways [29]. Moreover, addition of PI3K (LY294002), Akt (KP372-1), and ERK1/2 (PD98059) specific inhibitors completely blockaded the pro-proliferative effect of IGF-1 [14,19].

Our previous study also demonstrated that 15% mechanical stretch induced C2C12 myoblasts to secrete IGF-1, then increased PI3K/Akt and MAPK (p38 and ERK1/2) activities through the mediation of IGF-1R, and thus promoted the proliferation of C2C12 myoblasts [9]. In the present study, it was surprising to find that 15% stretch had no effect on inducing IGF-1 secretion of L6 myoblasts, which is totally different from C2C12 myoblasts. In addition, 15% stretch on L6 myoblasts enhanced the protein level of IGF-1R, similar to C2C12 myoblasts. Considering the important role of IGF-1R on IGF-1 signal, we speculated that 15% stretch-induced increases of PI3K/Akt and ERK1/2 activities in L6 myoblasts were mediated by IGF-1R, despite no detectable IGF-1 secretion. Further study is needed to thoroughly clarify the effect of IGF-1R on PI3K/Akt and ERK1/2 activations using IGF-1R specific inhibitor (such as picropodophyllin).

However, excessive stretch or overstretch inhibited cell proliferation. The only report about the anti-proliferation of overstretch was our previous study, which indicated that anti-proliferation of 20% stretch on C2C12 myoblasts was likely to be mediated by attenuated activations of PI3K/Akt, p38, and ERK1/2 [9]. In the present study, the same result was achieved, and indicated that anti-proliferation of 20% stretch on L6 myoblasts might be mediated by decreased activations of PI3K/Akt, p38, and ERK1/2, and no cell difference between L6 and C2C12 myoblasts. Furthermore, treatment with IGF-1 recombinant peptide reversed the proliferation inhibition of L6 myoblasts, accompanied with the increase of IGF-1R protein level, as well as the enhancements of PI3K/Akt, p38, and ERK1/2 activities, which indicated that 20% stretch-induced proliferation inhibition of L6 myoblast might be associated with the inhibitions of PI3K/Akt, p38, and ERK1/2 activities resulting from the decline of IGF-1R. 

There are some strengths and limitations of our study. Some new discoveries were reported: (1) it is the first report about the cyclic mechanical stretch on the proliferation of L6 myoblasts; (2) 15% stretch has no effect on the IGF-1 secretion of L6 myoblast and the pro-proliferation of 15% stretch is unrelated to p38 pathway, which are totally different from that observed in C2C12 myoblasts; (3) the stretch-induced proliferation alterations of L6 myoblast may be mediated by alterations in PI3K/Akt and MAPK activations regulated by IGF-1R, despite no detectable IGF-1 from stretched L6 myoblasts. The limitation of the study was short of the results about the influence of IGF-1R specific inhibitor on the activations of PI3K/Akt and MAPKs in 15% stretched L6 myoblast, so we failed to thoroughly verify the mediation of IGF-1R in 15% stretch-induced activations of PI3K/Akt and MAPKs.

In conclusion, 15% cyclic mechanical stretch promoted, while 20% stretch inhibited the proliferation of L6 myoblasts. The stretch-modulated proliferation was likely to be attributed to the changes of PI3K/Akt and MAPKs activations regulated by IGF-1R, despite no detectable IGF-1 from stretched L6 myoblasts. These results provide theoretical support for stretch-induced increase in skeletal muscle mass and overstretch-induced decrease in skeletal muscle mass.

## 4. Materials and Methods

### 4.1. Cell Culture

Rat L6 myoblasts were purchased from Chinese Academy of Sciences (Shanghai, China), and were maintained in Dulbecco’s modified Eagle’s medium (DMEM) (Gibco, Grand Island, NY, USA), containing 10% fetal bovine serum (Gibco, USA), and 100 U/mL penicillin and 100 µg/mL streptomycin, at 37 °C in a humidified atmosphere containing 5% CO_2_. L6 cells at low passages (P3 to P8) were used in all the experiments.

### 4.2. Cyclic Mechanical Stretch

The stretch model of L6 myoblasts in vitro was established using computer-controlled cell stretching equipment (Flexcell FX-5000TM Tension System, FlexCell International Corporation, Burlington, NC, USA). Briefly, L6 cells were seeded onto flexible-bottom six-well plates coated with type-I collagen (BioFlex, FlexCell International Corporation, USA) at a density of 1 × 10^5^ cells/mL in culture medium and incubated for 24 h. The cells were then subjected to cyclic strain (15% or 20% deformation) at 0.5 Hz (1 s of stretch, 1 s of relax) for 6 h. Cells cultured under the same conditions without any mechanical cyclic were considered as control.

### 4.3. Detection of Cell Proliferation

After stretch finished, culture medium was replaced with fresh medium and incubated for 24 h, then Cell Counting Kit-8 reagents (Dojindo Laboratories, Kumamoto, Japan) were added into the medium in proportion, followed by further incubation for 2 h. The optical density (OD) values were detected at 450 nm using a microplate reader (Biotek, Winooski, VT, USA). 

### 4.4. Determination of IGF-1 Concentration in Cell Culture Supernatant

After stretch finished, culture medium was immediately replaced using DMEM with or without fetal bovine serum, and incubated for 24 h, then the culture medium was collected and centrifuged at 12,000 rpm for 5 min at 4 °C. IGF-1 concentration of the supernatant was detected by IGF-1 ELISA Kit (Mouse/Rat IGF-1 Quantikine ELISA Kit, MG100, R&D Systems Inc., Minneapolis, MN, USA) according to the manufacturer’s instructions. 

### 4.5. Treatments of L6 Myoblasts with IGF-1 Recombinant Peptide, and Specific Inhibitors of PI3K, p38, and ERK1/2

To verify the role of PI3K, p38, and ERK1/2 in L6 myoblasts proliferation induced by cyclic mechanical stretch, different concentration (20, 40, or 60 μM) of specific inhibitors of PI3K (LY294002), p38 (203580), and ERK1/2 (U0126) were added into the culture medium and incubated for 2 h prior to 15% cyclic mechanical stretch. In order to testify the role of IGF-1 in the proliferation of L6 myoblasts regulated by cyclic mechanical stretch and its associations with PI3K/Akt and MAPKs protein levels and activities, IGF-1 recombinant peptide (26, 66, or 132 mM) was added into the medium before cells were subjected to 15% or 20% cyclic mechanical stretch.

### 4.6. Western Blot

Cells were lysed with RIPA buffer (Beyotime Biotechnology, Shanghai, China) containing protease and phosphatase inhibitors (Beyotime Biotechnology, Shanghai, China) at 24 h after stretch finished, and centrifuged at 12,000 rpm for 20 min at 4 °C, then the protein concentration was determined using enhanced bicinchoninic acid assay kit (Beyotime Biotechnology, China). Protein loading buffer (5×; Sangon Biotech, Shanghai, Shanghai, China) was added into the lysates prior to denaturation on bath incubator for 10 min. A total of 50 μg protein was electrophoresed on 10% SDS-PAGE gels, then transferred to polyvinylidene fluoride (PVDF) membrane and blocked in 5% non-fat milk for 2 h at room temperature, then incubated at 4 °C overnight with primary antibody, such as IGF-1R (AF-305, 1:500) (R&D Systems Inc.), PI3K (4257P, 1:1000), p-PI3K (p85 (Tyr 458)/p55 (Tyr 199), 4228S, 1:1000), Akt (4691P, 1:1000), p-Akt (Ser 473, 4060S, 1:1000), p38 (8690P, 1:1000), p-p38 MAPK (Thr 180/Tyr 182, 4511S, 1:1000), p44/42 MAPK (4695P, 1:1000), or p-p44/42 MAPK (Thr 202/Tyr 204, 4370P, 1:1000) (Cell Signaling Technology, Danvers, MA, USA). Then, the PVDF membranes were rinsed three times with TBST (5 min/wash) and incubated with horseradish peroxidase (HRP)-conjugated secondary antibodies for 2 h at room temperature, and subsequently, the membranes were rinsed three times as described above, prior to visualization by automatic chemiluminescence apparatus (Tanon Biotechnology, Shanghai, China), and the densities of bands were analyzed by Image J software.

### 4.7. Statistical Analyses

All the experiments were repeated at least three times, the data are expressed as mean ± standard deviation (SD). Statistical analyses were performed using one-way ANOVA and Bonferroni post hoc comparing by SPSS 21.0 (IBM Corporation, Armonk, NY, USA), and *p* < 0.05 was considered as a significant difference.
